# Radially Aligned Carbon Nanotube Glass Fiber Composites as Ion-Selective Microelectrodes

**DOI:** 10.1021/acsomega.4c07239

**Published:** 2025-02-14

**Authors:** Ahmet Önder, Zhi Kai Ng, Siu Hon Tsang, Palaniappan Alagappan, Edwin Hang Tong Teo, Ümit Hakan Yildiz

**Affiliations:** a Department of Chemistry, Izmir Institute of Technology, Urla, Izmir 35430, Türkiye; b Temasek Laboratories, Research Techno Plaza, 50 Nanyang Drive, Singapore 637553, Singapore; c School of Materials Science and Engineering, Nanyang Technological University, 639798, Singapore; d School of Electrical and Electronic Engineering, Nanyang Technological University, Singapore 639798, Singapore

## Abstract

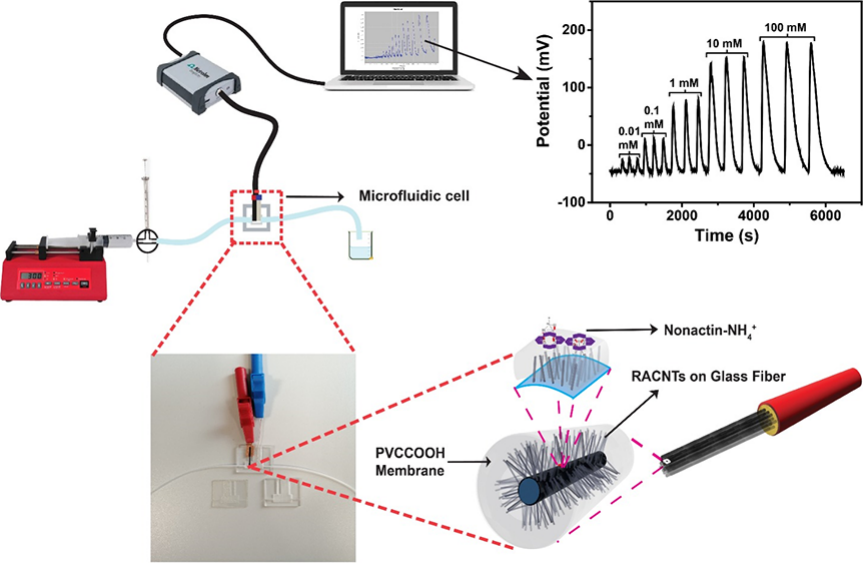

Detection of ions is challenging due to their small size, rapid diffusion, and high mobility, especially for assaying in samples of low volumes. Among the traditional analytical methods, potentiometric ion-selective electrodes (ISE) have become a popular choice for detecting ions as they are cost-effective, user-friendly and can be miniaturized, making them useful for on-site analysis. In this context, radially aligned carbon nanotubes (RACNT) directly grown on glass fibers (GF) via the chemical vapor deposition method is investigated as a solid contact material for the fabrication of ion-selective microelectrodes (μISE) upon incorporating specific ionophores within a polymeric encapsulation membrane. As an illustration, sensitive detection of ammonium ions is accomplished by the fabricated μISE (plasticized PVC membrane containing nonactin ionophores), which yielded a LOD and a linear response range between 7.5 × 10^–6^ and 1.0 × 10^–5^ to 1.0 × 10^–1^ M, respectively. The μISE fabricated with RACNT-GF as an interface material exhibited improvements in LOD and enhanced the detection selectivity as compared to a conventional ISE fabricated using planar solid contact materials such as graphite. We hypothesize that the fabricated μISE with a high surface area and mechanical durability maximize the accommodation of ionophores in the barrier membrane for yielding improved potentiometric responses. Experimental results illustrate that the μISE possesses the potential to be utilized for the fabrication of selective and sensitive ISE upon incorporation of specific ionophores with RACNT-GF composites.

## 1 Introduction

The detection of ions in environmental samples serves as an indicator of pollution levels and eutrophication of natural water. Accurate assaying of ions is crucial for environmental and health protection as they serve as markers for assessing the quality of natural waters and enzymatic byproducts in crucial physiological processes^1,2^ In the recent years, there has been a growing emphasis on the need to control environmental pollutants, leading to an increased interest in development of facile sensors for detecting ions.^3^ The primary analytical methods employed for assaying ions involve spectroscopic measurements, capillary electrophoresis, and chromatographic analyses coupled with mass spectrometry techniques such as HPLC and GC-MS.^4^ While these methods exhibit good selectivity and sensitivity, their utilization is constrained by their substantial costs and assay time. Furthermore, these sophisticated instruments may not be applicable for real-time or on-site analyses. Therefore, there is a need for the development of assays that are facile, cost-effective, and yield rapid assay responses.^4^ In this context, significant research emphasis has been devoted to the development of potentiometric ion-selective electrodes (ISE), owing to their potential for miniaturization.^1^ Potentiometric solid-contact ISE has been reported to provide numerous benefits over conventional liquid-contact ISE due to their ease of operation, compactness, portability, rapid response, and cost-effective fabrication.^5^ Typically, solid contact electrodes are fabricated using polymer materials such as epoxy, incorporated with conducting materials such as graphite,^6,7^ macroporous carbon, spherical carbon,^8^ C60 fullerenes, carbon nanotubes (CNT),^9,10^ carbon fiber,^11,12^ highly ordered pyrolytic graphite,^13^ etc. The conducting material serves as an interface for establishing and maintaining the electrical contact between the sample solutions and the ISE’s transduction elements, whereas the polymer materials provide the robustness for the ISE.^14−24^ Thus, the performance of potentiometric ISE is significantly influenced by the morphological and electrical properties of both the polymeric and the conducting materials utilized for their fabrication.

Among the conducting materials evaluated for fabrication of ISEs, the high aspect ratio and surface area of CNT accompanied by their high electrical conductivity and thermal and electrochemical stability^25^ render them appealing for their utilization as solid contact materials in electrochemistry. CNT have been widely reported to enhance the performance of various electrochemical devices such as sensors, supercapacitors, fuel cells, lithium-ion batteries, and electrocatalysis.^26−28^ This ion-to-electron transduction is primarily driven by the formation of an asymmetric capacitor, where one side is composed of charge carriers in the CNT walls while the other is formed by the ions in the solution, enabling effective charge coupling at the CNT/electrolyte interface. Further, the hydrophobic CNT with a large double-layer capacitance prevent water layer formation ensuring consistent, long-term analytical performance, making them a reliable interface material for the fabrication of solid-contact ISEs.^29^ In addition, utilization of aligned CNT has been reported to be beneficial for the development of selective and sensitive electrochemical assays.^30^ In this context, glass fiber (GF), a material that is robust and cohered of defined GF filaments with diameters in the order of few microns, could further improve the surface area for the deposition of CNT, potentially enabling the combination of GF and CNT to be an ideal composite for the fabrication of sensitive ISE. Herein, CNT are directly grown on GF via a chemical vapor deposition (CVD) method and are evaluated as a solid contact material for the fabrication of potentiometric ion-selective microelectrodes (μISE). The adopted fabrication protocol yielded a composite with CNT aligned radially on individual GF filaments. The radial alignment of the CNT and the fibrous structure of the GF provides a high surface area for maximizing the accommodation of the ionophores within the polymer membrane, improving electrical contact, enhancing stability, and increasing durability. The specificity and sensitivity of the fabricated μISE are then validated by the detection of ammonium ions (NH_4_^+^), an ion of significant assaying interest, by incorporating RACNT with NH_4_^+^ selective nonactin ionophore within bis(2-ethlhexyl) sebecate (DOS) plasticized polyvinyl chloride (PVC) membranes. The capability of utilizing μISE for detection of ions in small volume samples is subsequently investigated by performing the assay in a microfluidic cell. The compatibility of the μISE with standard microfabrication techniques enables the development of low-cost and disposable sensors for applications such as water quality monitoring, disease diagnosis, and assessing agricultural soil health. Furthermore, the potential integration of the μISE with microfluidics could enable accurate detection of ions in small volume samples.^31−36^

## 2 Materials and Methods

### 2.1 Materials

Analytical-grade tetrahydrofuran (THF), nonactin, high-molecular-weight PVC, carboxylated PVC (PVC–COOH), *o*-nitrophenyl octyl ether (NPOE), dioctyl phthalate (DOP), DOS, aluminum nitrate (Al(NO_3_)_3_), iron nitrate (Fe(NO_3_)_3_), and all the reagents used for the synthesis of CNT were procured from Sigma-Aldrich. Distilled deionized (DI) water was used for all the experiments. The gases for CNT growth were purchased from National Oxygen, Singapore.

### 2.2 Growth of CNT on GF

**Figure 1:**
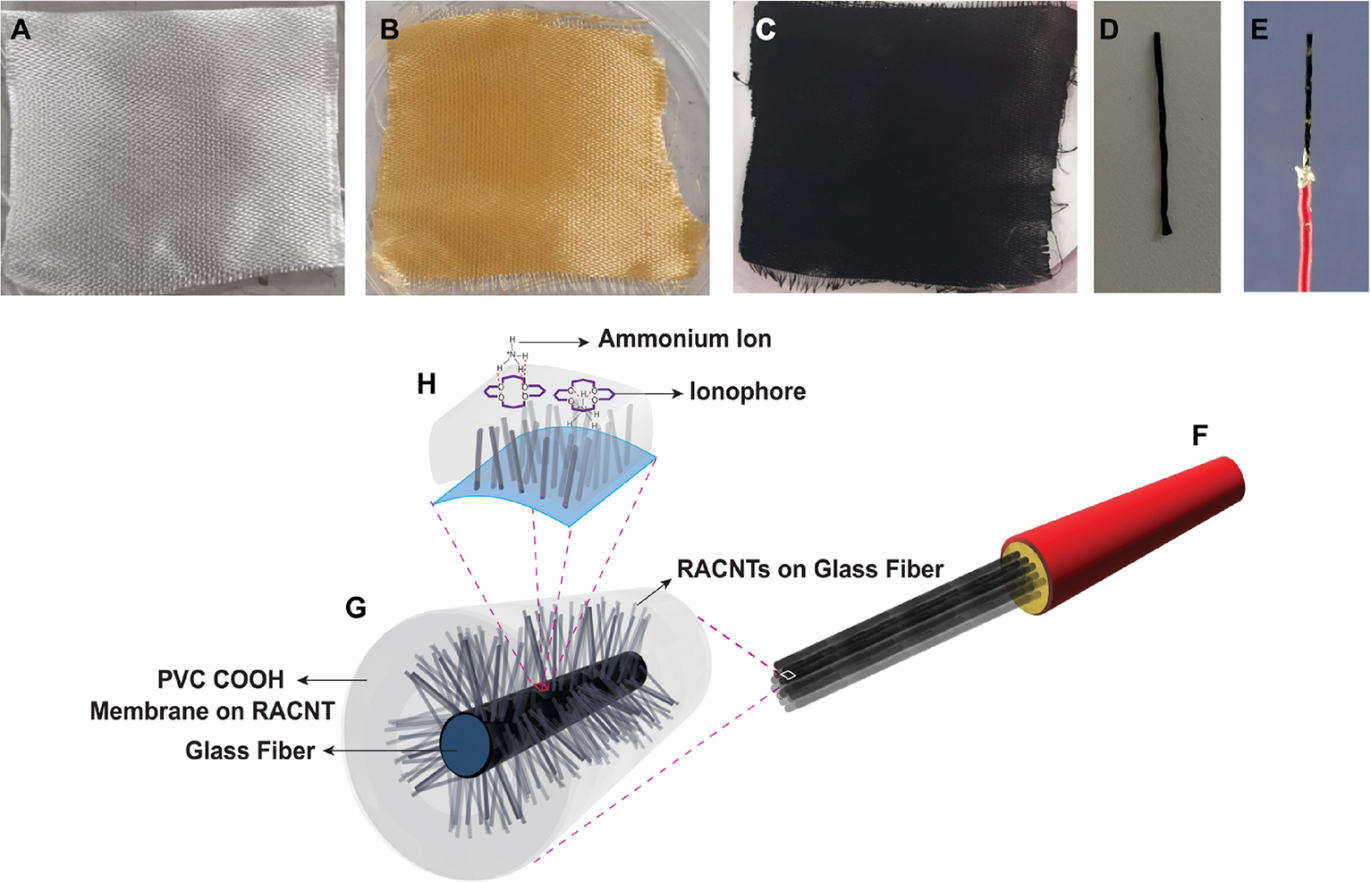
As-procured 10 cm × 10 cm GF fabric (A) dip-coated with an Fe:Al catalyst (B) for subsequent growth of CNT (C). (D) Individual GF fibers with a homogeneous RACNT growth in the radial orientation subsequently soldered to a copper wire and ensheathed using an insulator for fabrication of the μISE. The schematic of the μISE (F) illustrates the radial alignment of CNT on GF within the PVC–COOH membrane (G) and incorporation of nonactin for selective detection of NH_4_^+^ (H).

GF fabrics procured from Hexcel (Hexforce 1543) were cut into 10 cm × 10 cm sizes (Figure 1A). For CNT growth catalyst deposition, Al(NO_3_)_3_ and Fe(NO_3_)_3_ were chosen as the Fe and Al salts, respectively, due to their solubility in water. 100 mL of DI water was mixed with the salts, with varying Fe:Al salts ratios (1:2, 1:10, and 1:50). The GF fabric was then dip-coated with catalyst solutions (Figure 1B) followed by a CVD growth process to grow the CNT on the GF. In brief, the GF fabric containing the catalyst was placed in the CVD furnace (FirstNano EASYTUBE 3000). The temperature is increased to 750 °C over 60 min under Ar and H_2_ flow at a ratio of 3:1. C_2_H_4_ and Ar (via H_2_O bubbler maintained at 50 °C) were then introduced into the chamber with a ratio of 24:8:6:1 (Ar:H_2_:CH_4_:H_2_O). After 30 min of CNT growth, the C_2_H_4_ and H_2_O flow were cut off and the chamber is allowed to cool. H_2_ was cut off at 400 °C, and the GF with RACNT (Figure 1C) was removed from the chamber at room temperature. The obtained RACNT-GF composite was then characterized using SEM and Raman spectroscopy. The other characteristics of the RACNT-GF are elaborated in the Supporting Information.

### 2.3 Fabrication of the μISE

Individual GF fibers from the GF fabric deposited with RACNT are dislodged for the fabrication of the μISE (Figure 1D). Each fiber in the fabric is composed of a defined number of GF filaments: 816 and 204 filaments with diameters of 9 and 7 μm, in the vertical and horizontal directions, respectively (Hexcel 1543 fabric). The individual RACNT-GF composite fiber is then dip-coated in THF solution consisting of 100 mg of PVC/PVC–COOH, plasticizers, and nonactin in varying stoichiometric ratios (Figure 1E). The μISE is schematically illustrated in Figure 1F–H, showing the surface area enhancement via radial alignment of CNT on fibrous GF for maximizing the incorporation of nonactin, thereby yielding the selective and sensitive detection of NH_4_^+^. A pencil graphite electrode (PGE) with 0.7 mm diameter was utilized for control experiments. The PGE was treated as reported previously.^37,38^ The PGE is then dip-coated in the solution containing the optimum membrane composition (PVC–COOH (∼30%), DOS (67%), and nonactin (3%)). Herein, PGE with the same mass equivalent of μISE was utilized to compare the responses of these two electrodes with planar and radially aligned carbon morphologies. The total carbon contents of μISE and PGE were standardized gravimetrically for all the control experiments.

### 2.4 Potentiometric Setup and Measurement

Potentiometric measurements were performed using the μStat-i 400s instrument, a Metrohm potentiostat/galvanostat equipped with a high-impedance electrometer designed for precise measurements. The steady-state measurements were obtained by submerging the μISE (as the working electrode) and a Ag/AgCl reference electrode (filled with saturated potassium chloride (36% w/w)) to an equal depth in a 30 mL assay solution while ensuring continuous stirring at a constant rate. Before each measurement sequence, both the reference and μISE were rinsed with DI water and carefully dried by using a lint free tissue. The μISE were conditioned by soaking into 0.01 M NH_4_NO_3_ solution for 4 h before the measurements. The potentiometric responses were then recorded for NH_4_^+^ as well as other monovalent cationic species by immersing the μISE in the corresponding standard solutions with concentrations ranging from 1.0 × 10^–6^ to 1.0 × 10^–1^ M.

### 2.5 Flow-Injection Analysis Setup

**Figure 2:**
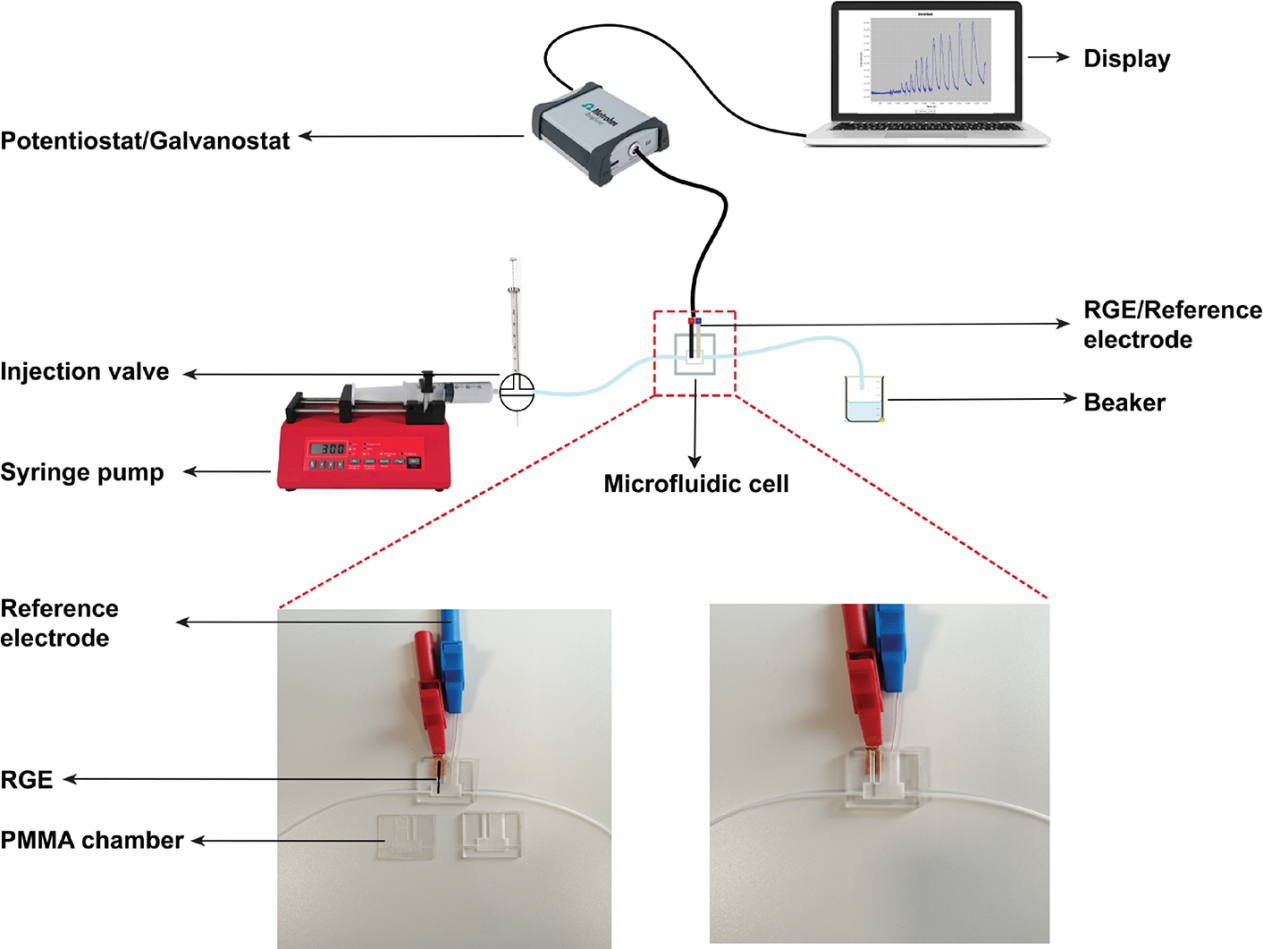
Flow injection setup to evaluate the performance of the μISE.

A flow-injection analysis (FIA) setup consisting of a microfluidic cell, a syringe pump, and a valve was utilized for the quantification of NH_4_^+^ in small volume samples. The cell was fabricated using a laser cutter to create the microfluidic channels and grooves for accommodating the RACNT-GF composite on the PMMA substrates with dimensions of 2 cm × 2 cm and thickness of 3 mm. The reference electrode (Ag/AgCl (60/40) paste with Ag wire) and the μISE (working electrode), both with a diameter of approximately 0.5 mm and length of 2.0 cm, were positioned within the slots on the PMMA substrate as illustrated in Figure 2. Another PMMA substrate was then utilized to seal the cell using a double-sided tape to ensure a secure bonding. 60 μL of standard ammonium solution in a carrier solution of 1.0 mM NaNO_3_ was injected at a flow rate of 0.5 mL/min via an injection loop. The Metrohm potentiostat/galvanostat was used to monitor potentiometric responses via the DropView software. The reference electrode in the proposed design operates as a pseudo-reference electrode, which is without an internal saturated KCl solution. Although this reference electrode enables miniaturization, it is to be noted that the lack of a saturated KCl solution restricts potential stability in samples with ion concentrations above 1.0 × 10^–2^ M, especially for chloride ions. Potential stability measurements of reference electrodes are provided in the Supporting Information (Figure S11).

## 3 Results and Discussion

### 3.1 Morphological Characterization

**Figure 3:**
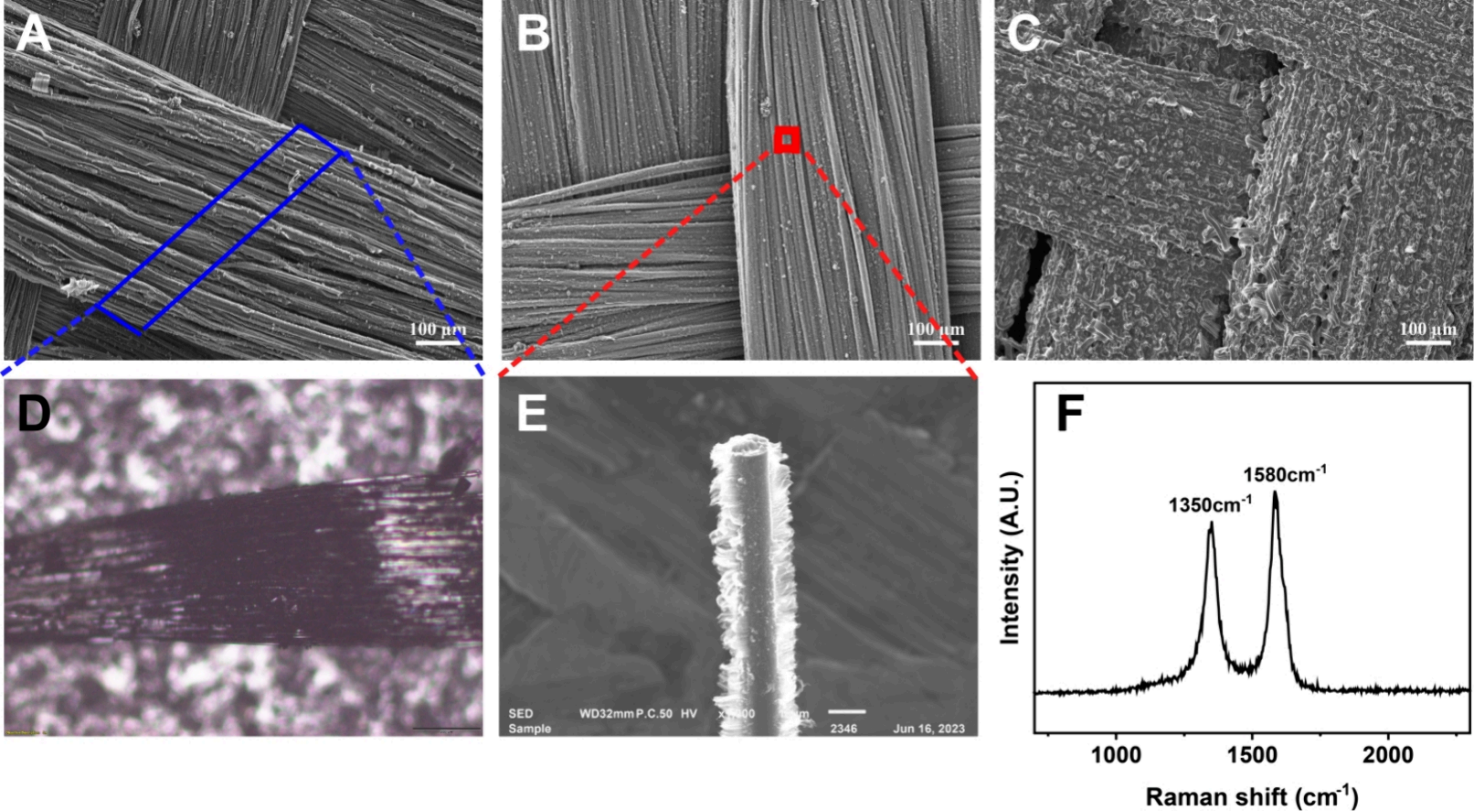
GF with CNT grown using three different Fe:Al catalyst ratios of 1:2 (A), 1:10 (B), and 1:50 (C). Optical microscopy image of individual GF fibers after CNT growth (D), SEM image of individual GF filaments after CNT growth showing radial alignment of CNT (E), and the Raman spectrum of CNT on the GF fabric (F).

As-procured, pristine GF fabric shown in Figure 1A was dip-coated with different catalyst ratios (1:2, 1:10, and 1:50) by immersing them in the Fe:Al catalyst solutions. Various parameters such as number dip-coating cycles and rate of withdrawal of GF fabric were optimized to obtain a uniform coating of catalyst on the GF fabric, as illustrated by the homogeneous pale yellow color observed in Figure 1B. CNT were subsequently grown directly on the GF fabric using a CVD growth protocol in which all the growth parameters, such as growth time, temperature, etc., were optimized to yield a homogeneous distribution of CNT on the GF fabric as shown in Figure 1C. The three different catalyst ratios yielded CNT growth, which can be categorized into 3 morphologies based on SEM observations: radially aligned and nonhomogeneous (Figure 3A), radially aligned and homogeneous (Figure 3B), and random orientation and nonhomogeneous (Figure 3C). The improvement in CNT homogeneity and radial alignment for the Fe:Al catalyst ratio of 1:10 could be attributed to the optimal ratios of aluminum salts that prevents the aggregation of large Fe nanoparticles, allowing for optimal distribution of Fe on the GF fabric for CNT growth.^39^ It is hypothesized that minimal or excess aluminum salts either yields nonhomogenous Fe nucleation sites on the GF fabric or prevents the carbon precursor from reaching the Fe nucleation sites, thereby inhibiting CNT growth. Figure 3D shows the optical microscopy image of individual GF in the GF fabric (highlighted in the blue box in Figure 3A) consisting of cohered GF filaments homogeneously deposited with CNT. An individual GF filament (highlighted in the red box in Figure 3B) examined under a high-resolution SEM reveals a darker core and a lighter shell (Figure 3E), corresponding to the GF filament and RACNT, respectively, further ascertaining the radial alignment of CNT and their homogeneous growth on GF filaments. The diameter of individual fiber is ∼10 μm, which concurs with the specification of the as-procured GF fabric. Raman spectroscopy of the GF/CNT shows the signature D (1350 cm^–1^), G (1580 cm^–1^), and 2D (2680 cm^–1^) peaks of carbon nanomaterials. The ratio of D to G peak is ∼ 0.8:1, which is typical of CVD-grown CNT. An anisotropy of the G peak is observed, which indicates that the CNTs are multiwalled in nature (Figure 3F).^40−43^ An Fe:Al ratio of 1:10 is thus determined to be optimal for fabrication of the μISE, where the GF and RACNT could collectively maximize the surface area for effective interaction with assay solutions. The detailed protocols for obtaining a homogeneous growth of RACNT on the GF are provided in the Supporting Information (Figures S1 to S6). Contact angle measurements and XPS analysis for the RACNT and PGE ISE are also provided in the Supporting Information (Figures S9 and S10).

### 3.2 Optimization of Electrode and Ionophore Compositions

**Figure 4:**
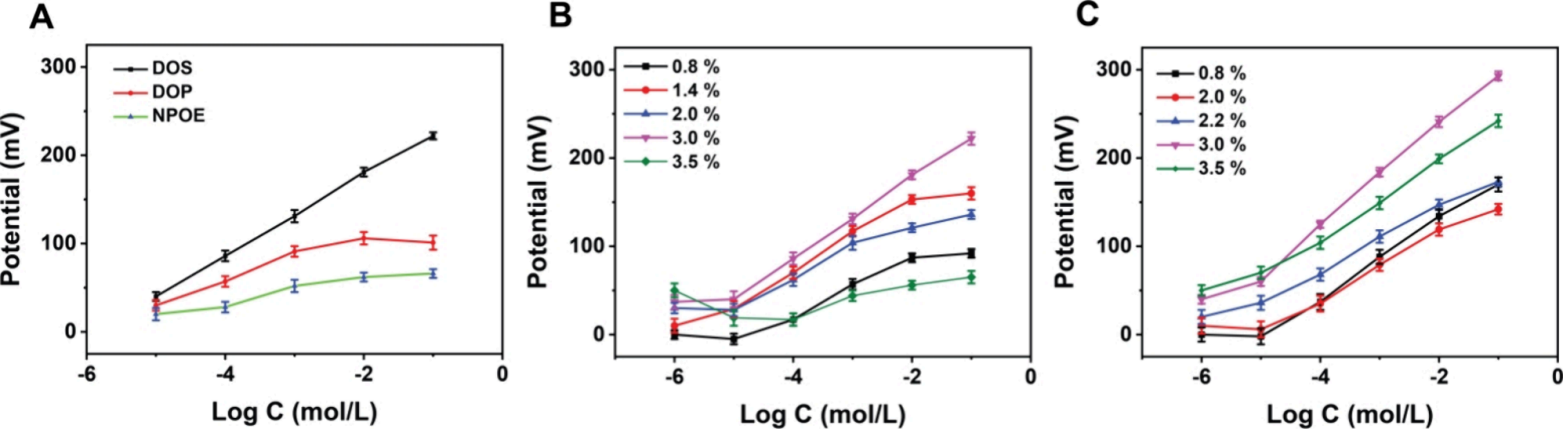
Potentiometric response of μISEs to varying concentrations of NH_4_^+^ prepared using different plasticizers (A) (with constant nonactin concentration (3%)) and increasing concentrations of nonactin (with PVC membrane (B) and PVC–COOH membrane (C)).

PVC has been reported to be a potential membrane for the fabrication of ISE owing to its chemical stability. However, the sensitivity, linearity, and selectivity of electrodes are significantly influenced by the composition of the polymeric membrane and plasticizers that accommodate the ionophore.^44^ Hence, optimization experiments were performed in which the RACNT-GF composite was incorporated with PVC/PVC–COOH membranes and three different plasticizers: DOS, NPOE, and DOP. As summarized in Table S1 (see SI), potentiometric responses were obtained for the μISE fabricated using varying concentrations of three different plasticizers at a fixed nonactin concentration (3%) and optimized ∼1:2 mass ratio of PVC:plasticizer.^1^Figure 4A reveals that the μISE yields a linear response with DOS as compared to DOP and NPOE plasticizers. DOS with lower polarity could facilitate the diffusion of ions and effectively distribute them within the membrane. Subsequently, the influence of membrane composition was investigated using two membranes: PVC and PVC–COOH with DOS as the plasticizer at varying nonactin concentrations. Figure 4B,C illustrates that the most favorable potentiometric characteristics in terms of linear range and slope are obtained for the PVC–COOH membrane with a nonactin concentration of ∼3%. The major reason for the increased response could be attributed to the presence of negatively charged COOH/COO groups in the membrane. In accordance with the Donnan exclusion effect, the membrane with a negative charge would allow the cations to pass through more easily than anions, facilitating nonactin-NH_4_^+^ interactions. It should be noted that increasing the concentration of ionophores from 3.0% to 3.5% could negatively influence the potentiometric responses owing to the saturation of the ISE membrane with ionophores. As observed from Table S1, PVC–COOH (∼30%), DOS (67%), and nonactin (3%) are the optimal materials (and stoichiometric ratios, highlighted in bold) that can be deposited on μISE for sensitive detection of NH_4_^+^. The optimized ratio of nonactin within the appropriate plasticizer yielded NH_4_^+^ concentration dependent responses with improved detection sensitivity.

### 3.3 Selectivity, Sensitivity, and LOD Analysis

**Figure 5:**
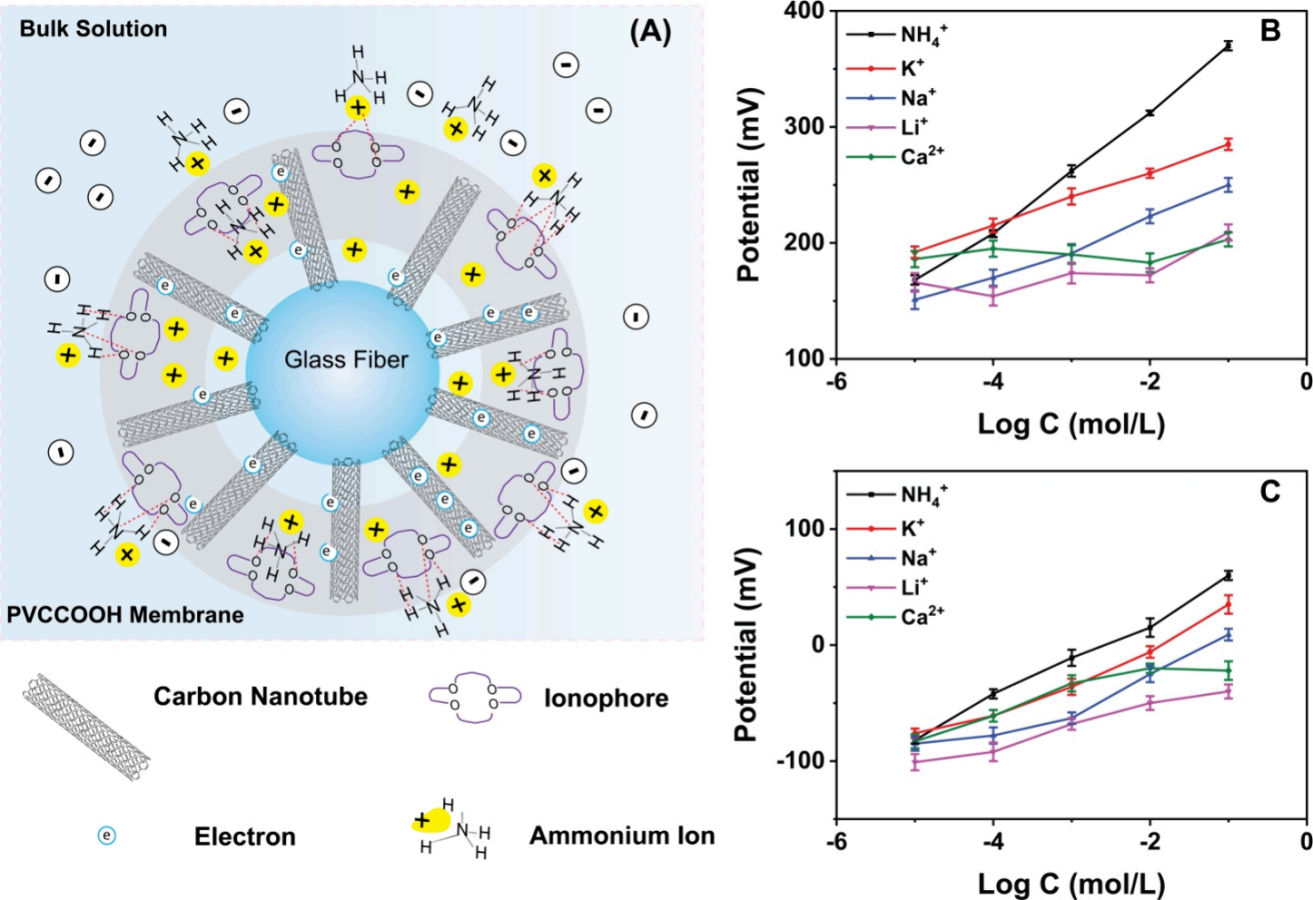
Sensing mechanism of the μISE (A) and potentiometric responses of (B) μISE and (C) PGE for varying concentrations of cations.

As illustrated in Figure 5A, the solid contact layer of the μISE consists of RACNT radially aligned on individual GF. Owing to the high surface area, utilization of RACNT as an interface material ensures efficient electrostatic gating and detection sensitivity via accumulation of NH_4_^+^ ions as compared to PGE with a planar morphology (Figure 5B,C).^29^ The potential stabilities of NH_4_^+^ selective RACNT and PGE were measured over 6 h. The RACNT electrode showed a gradual reduction in slope at −1.4 mV/h, indicating a relatively stable potential behavior, while the PGE displayed a rapid reduction at −3.8 mV/h, indicating a degradation in detection sensitivity (Figure S7). The lifetime of the RACNT ISE was estimated to be ∼15 days, and long-term studies are required to determine their accurate lifespan under varying assay conditions (Figure S8).

The potentiometric selectivity of an ISE holds significant importance as it illustrates the relative response of the ISE toward the primary ions in the presence of interfering ions. Conventionally, the determination of selectivity coefficients is performed using the methods outlined in the IUPAC protocol (the separate solution method was utilized to calculate the selectivity coefficients for various interfering ions).^45^ The procedure suggests a calibration curve using primary ion (NH_4_^+^ denoted as “*a*_A_”) solutions with concentrations ranging from 1.0 × 10^–6^ to 1.0 × 10^–1^ M. Subsequently, the μISE is tested in individual interfering ion solutions with a concentration of 1.0 × 10^–2^ M (denoted as “*a*_B_”), and the corresponding potential values of each interfering ions are recorded. Finally, the potential values are utilized to determine the corresponding activity of the primary ion using the calibration plot. The selectivity coefficient values shown in Table 1 were obtained by substituting the calculated primary ion activity (*a*_A_) and the corresponding interfering ion activity (*a*_B_) in the equation
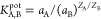
where *Z*_A_ and *Z*_B_ are net charges of primary and interfering ions.

**Table 1:** Selectivity Coefficient Values for NH_4_^+^-Selective Electrodes Calculated by the Separate Solution Method (SSM)

interferents	–*K*_NH4,B_^pot^
K^+^	1.8
Na^+^	2.8
Li^+^	4.2
Ca^2+^	4.5

Table 1 shows that the tested cations do not cause significant interference and that the μISE maintains a selectivity of at least 100-fold, even when the most interfering ion, K^+^, is present in the assay solution. The selectivity of the μISE was compared with previous reports based on the solid contact types and their respective K^+^ selectivity, expressed through selectivity coefficients (Table S2).

**Figure 6:**
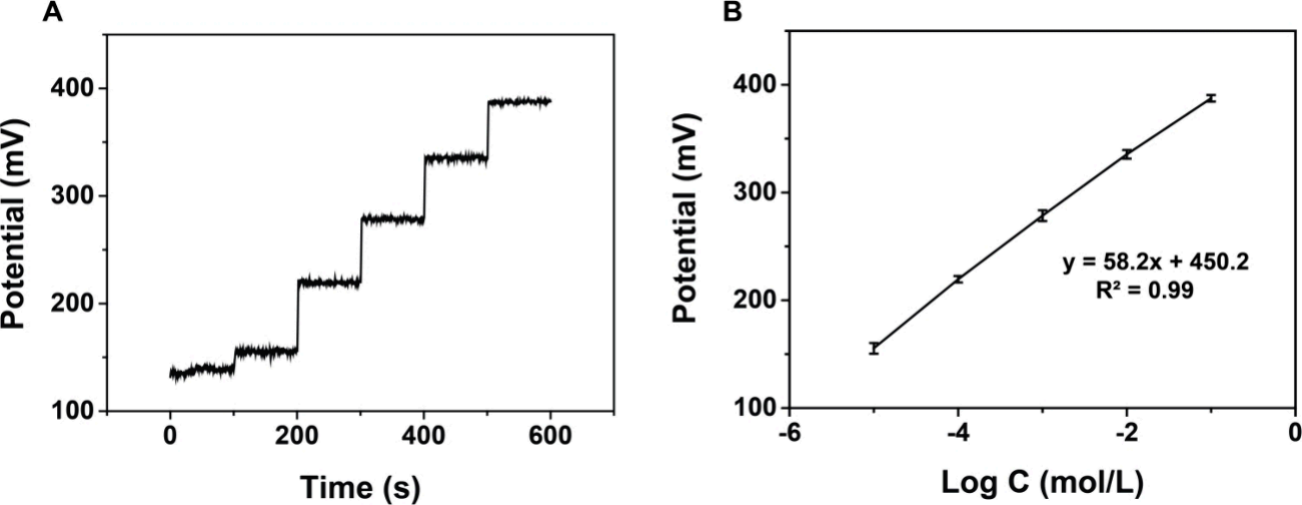
(A) Time-dependent potentiometric response with varying NH_4_^+^ concentrations and (B) linear calibration plot for NH_4_^+^ detection.

Figure 6A shows the time-dependent potentiometric response of the μISE to NH_4_^+^ ions obtained in aqueous solutions with NH_4_^+^ concentrations ranging from 1.0 × 10^–6^ to 1.0 × 10^–1^ M. As observed from the potential-log *C* plot (Figure 6B (concentrations not within the linear range, below 1.0 × 10^–6^ M, are not included)), the μISE yielded a linear response within the concentration range of 1.0 × 10^–5^ to 1.0 × 10^–1^ M (*R*^2^ = 0.99), exhibiting a slope of 58 ± 0.6 mV per order of concentration. Following the IUPAC recommendation,^46^ the LOD of the μISE was calculated as 7.5 × 10^–6^ M, obtained from the intersection of the two extrapolated sections of the calibration curve.

### 3.4 Evaluation of μISE Assay Performance in Small Volume Samples

**Figure 7:**
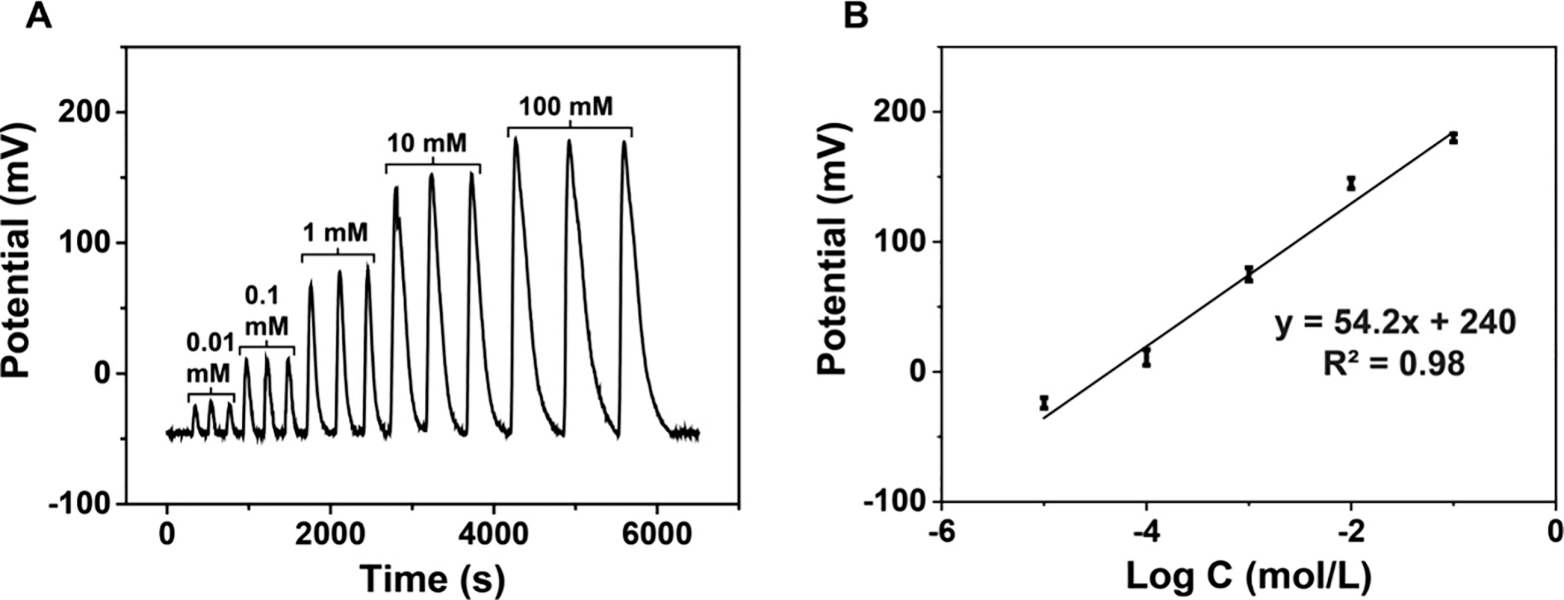
(A) Potentiometric response of the μISE in the FIA system and (B) calibration curve.

Figure 7 illustrates the typical flow injection responses observed for the μISE for repeated injections of a standard ammonium solution. The potentiometric assay consistency and the reproducibility of ±1.8 mV dec^–1^ in the microfluidic device could be observed for the triplicate injections of NH_4_^+^ for all the test concentrations. An increase in peak width was noted upon injecting higher concentrations, which could be attributed to the reduced rate of diffusion of NH_4_^+^ within the PVC–COOH membrane and could be optimized by controlling the injection flow rate of the assay solution. When operated in the FIA mode with a 1.0 mM sodium nitrate carrier solution, a near Nernstian response with a slope of 54.2 ± 1.2 mV dec^–1^ was obtained. The calibration curve shown in Figure 7B was derived from the flow injection potentiometric responses (Figure 7A) by averaging the responses obtained for triplicate injections. Linearity of the calibration curve illustrates that the effect of diffusion limitation and interference of carrier phase are not substantial. Although the μISE incorporates RACNT with transduction materials on the nanometer scale, the high density and uniform distribution of these materials minimize the diffusion-related limitations typically encountered in confined flow environments. The resultant slope of the μISE potentiometric response in microfluidic devices is identical to that of the slope obtained for measurements performed under steady-state conditions. Thus, the RACNT-GF composite could be an ideal candidate for the fabrication of μISE with miniaturized dimensions and robustness for assaying ions in microfluidic environments.

## 4 Conclusions

RACNT directly grown on GF via CVD were utilized to develop NH_4_^+^ selective ISE. For the fabrication of the μISE, the RACNT-GF composite surface was modified by coating with a PVC–COOH/DOS plasticized membrane containing nonactin at optimal stoichiometric ratios. The μISE yielded potentiometric characteristics: slope, LOD, and linear response range of ∼58.2 ± 0.6 mV dec^–1^, 7.5 × 10^–6^ M, and 1.0 × 10^–5^ to 1.0 × 10^–1^ M, respectively. Future studies shall focus on tuning the membrane composition, for instance, by the incorporation of lipophilic anions with the μISE followed by evaluation of their potentiometric responses via the protocols described in the manuscript. The feasibility of utilizing μISE for detection of ions in small volume samples in microfluidic devices is demonstrated by performing the assay in a microfluidic cell. μISEs in the FIA system yielded near-Nernstian response with a slope of 54.2 ± 1.2 mV dec^–1^ and LOD of 1.0 × 10^–5^ M. The μISE can be easily fabricated in various shapes and sizes, facilitating miniaturization and construction of microfluidic devices for multiplexed detection of various ions. Furthermore, the 10 cm × 10 cm GF fabric potentially yields a large number of ISEs in a single CVD growth process, enabling their mass production while significantly reducing the fabrication costs per ISE.

## Supporting Information Available

Fabrication of RACNT, contact angle measurements, SEM and Raman spectroscopy analysis, lifetime of electrodes, XPS analysis, and stability of reference electrodes (PDF)
