# Supplementary material for: Radially Aligned Carbon Nanotube Glass Fiber Composites as Ion-Selective Microelectrodes
Source: ACS Omega. 2025 Feb 14;10(7):6578–85. doi: 10.1021/acsomega.4c07239 (PMC11866012; doi:10.1021/acsomega.4c07239)
Supplement: Supplementary file 1 — ao4c07239_si_001.pdf [file ao4c07239_si_001.pdf]

SUPPORTING INFORMATION

Radially Aligned Carbon Nanotube Glass Fiber Composites as Ion-Selective Microelectrodes

Ahmet Önder [a], Zhi kai Ng [b], Siu Hon Tsang [b], Alagappan Palaniappan [c],
Edwin Hang Tong Teo [c,d*], Ümit Hakan Yildiz [a,*]
[a] Department of Chemistry, Izmir Institute of Technology, Urla, 35430, Izmir, Türkiye
[b] Temasek Laboratories, Research Techno Plaza, 50 Nanyang Drive, Singapore 637553, Singapore
[c] School of Materials Science and Engineering, Nanyang Technological University, Singapore, 639798
[d] School of Electrical and Electronic Engineering, Nanyang Technological University, Singapore 639798, Singapore.
E-mail: hhteo@ntu.edu.sg ;hakanyildiz@iyte.edu.tr

Parametric study of factors that influence the growth of RACNT on GF

A parametric study of factors that influence the growth and density of RACNT of GF was conducted. Apart from catalyst ratio discussed in the manuscript, the RACNT quality was investigated by varying the process parameters: annealing temperature, withdrawal rate of GF from catalyst solution, number of dip-coating cycles, growth temperature and growth duration. Each parameter is investigated in detail to determine a parameter set for the optimal growth of RACNT on GF.

GF annealing temperature

The surface hydrophilicity of the GF is an important parameter which determines the homogeneous deposition of the aqueous catalyst solution. The hydrophilicity of GF was improved by annealing in air. The influence of annealing temperature on the hydrophilicity of the GF was then evaluated via contact angle measurements. As observed from Fig. S1, the as procured GF surface appears to relatively more hydrophobic with a contact angle of ~120 ° than the GF annealed at temperatures over 200 °C.

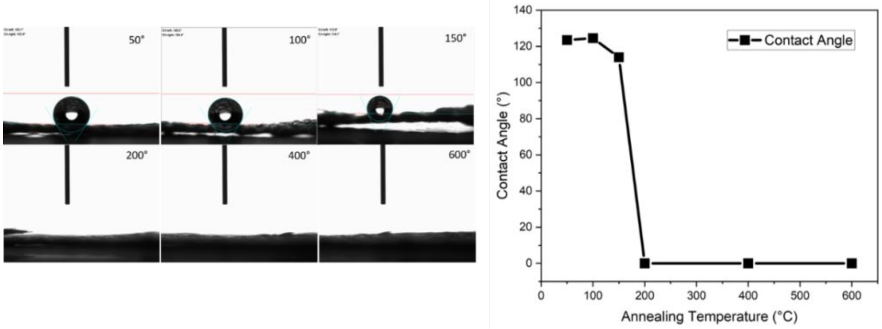

Figure S1: Contact angle of GF annealed at different temperatures (left) and the corresponding plot (right)

The contact angle versus temperature plot shows the abrupt transition of GF surface hydrophilicity at annealing temperature over ~ 175 °. Therefore, it is concluded that annealing at 200 °C would be sufficient to effectively hydrophilize the GF surface. It is hypothesized that the layer of protective coating on GF is effectively burned away at an annealing temperature of 200 °C, thus exposing the underlying hydrophilic silica layer.

Figure S2 shows that for the non-annealed GF, RACNT grows in the ridges between the individual GF strands, which could be due to the accumulation of the Fe catalyst in between the GF strands upon drying of the GF. There is no observable growth of RACNT on the surface of the GF strands. However, for the annealed GF, RACNT grows more homogenously and denser, indicating that the Fe catalyst has been uniformly deposited on the surface of the individual GF strands, which serve as nucleation sites for the uniform RACNT growth. Hence, annealing of GF at 200 °C is critical for conformal catalyst deposition of GF using the proposed dip-coating technique.

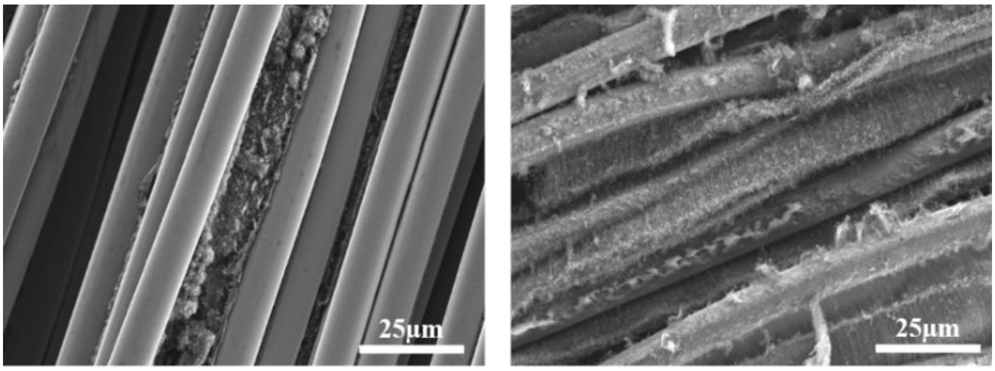

Figure S2: SEM images of the RACNT on non-annealed (left) and annealed (right) GF

Rate of withdrawing glass fabric from catalyst solution

The withdrawal rate of substrate upon dipping in the catalyst solution influences catalyst deposition of the intended substrates [1]. Herein, RACNT growth is evaluated using 2 withdrawal rates, differentiated as “fast pull (120 cm/min)” and “slow pull (~2 cm/min)”. RACNT growth using the “fast pull” method is sparse (red circle) as compared to the aligned RACNT growth (yellow circle) using the “slow pulling” method. These observations suggest that “slow pulling” enables uniform deposition of Fe catalyst and hence adopted for the homogeneous growth of RACNT on GF .

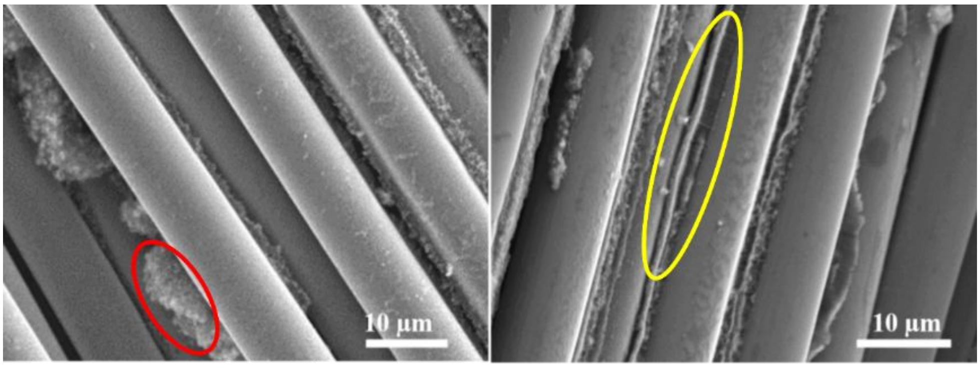

Figure S3: SEM images of as-grown RACNT using catalyst deposition by “Fast pull” (left) and “Slow pull” (right).

Number of dip-coating cycles

Multiple dip-coating cycles were performed to investigate the improvements in catalyst deposition. The number of dip-coating cycle: (i) soaking of GF annealed at 200 °C in catalyst solution for 3 min, (ii) withdrawal of GF using the "slow pull" method, (3) drying on a hotplate at 80 °C for 5 min a side, is varied (1, 3 and 5 times) to investigate the RACNT growth.

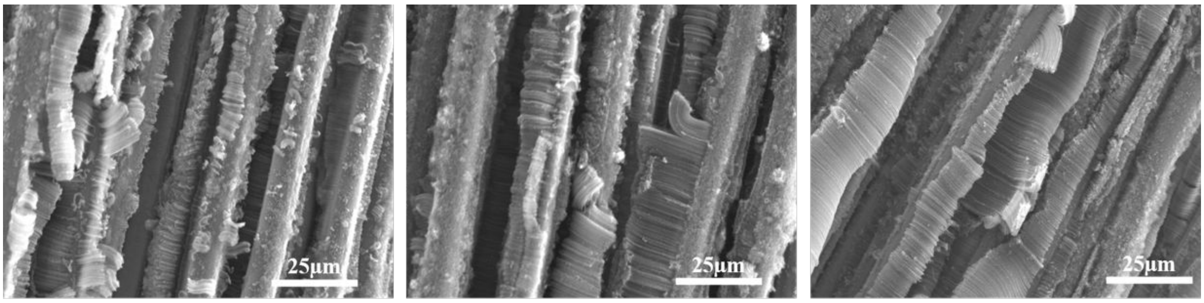

Figure S4: SEM image of as-grown RACNT after 1 (left), 3 (middle) and 5 (right) dip-coating cycles.

As observed from Fig. S4, there is no difference in the density and length of the as-grown RACNT using different number of dip-coating cycles. Since annealing of GF at 200 °C yield a hydrophilic surface, no further enhancement in the amount of catalyst deposition on GF could be achieved through multiple dip-coating cycles. It is concluded that the number of times to dip-coat does not significantly affect the RACNT growth on hydrophilized GF. Herein, 3 cycles of dip-coating were carried out to ensure repeatability and homogeneous catalyst deposition on GF.

Growth temperature

Growth temperature affects many aspects of RACNT growth including its crystallinity due to the effective breakdown of carbon precursors. The quality of the RACNT grown directly on GF at growth temperatures between of 650 and 750 °C was investigated using Raman spectroscopy by extracting the ratio of intensity of the D peak against the G peak (ID/IG).

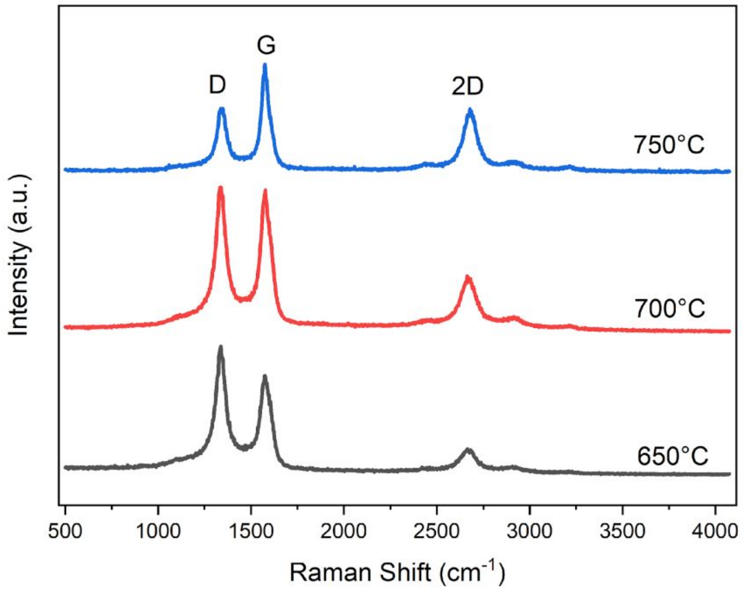

Figure S5: Raman spectra of the RACNT grown at 650, 700 and 750 °C.

The intensity of a D peak signal is an indicative of the concentration of defects in the RACNT [2,3]. Since the D peak is corelated to the concentration of defects within the RACNT, a lower I_D/I_G ratio would indicate a less defective RACNT. As shown in Fig. S5, there is a decrease in the I_D/I_G ratio with the increase growth temperature, which could be attributed to the complete decomposition of the carbon precursors resulting in reduced defect density of the RACNT. Therefore, a growth temperature of 750 °C was adopted for the homogeneous growth of RACNT on GF. It should be noted that the obtained Raman spectra is comparable to that of multi-walled RACNT in previous report [4], which was also synthesized at 750°C.

Growth time

The influence of RACNT growth time on RACNT growth was then investigated. As observed from Fig. S6, as the growth time increases, the GF appears darker, which is an indicative of longer RACNT growth. RACNT length after growth for 20 min is around a few µm, and the average length increases to ~ 10 µm (Fig. 3E in the manuscript) at a growth time of 30 min. At a growth time of 40 min, there is no observable change in the RACNT length as compared to the growth time of 30 min, indicating that the growth rate of RACNT saturates after 30 min. Therefore, a growth time of 30 min was utilized for the fabrication of GF-RACNT composite.

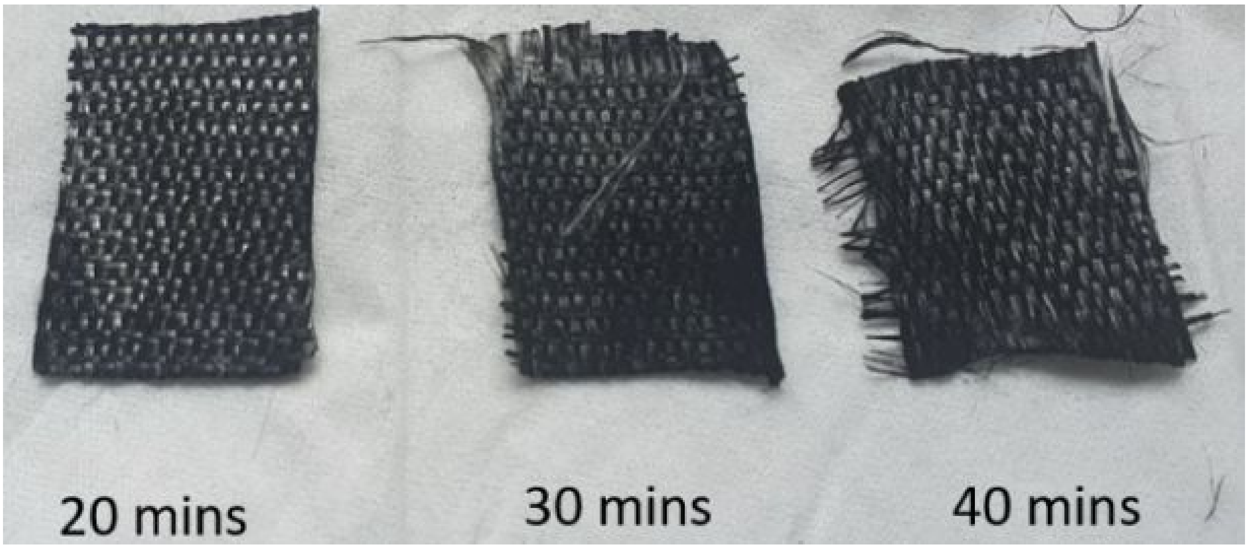

Figure S6: The influence of growth time on GF/RACNT

Optimization of membrane compositions

In PVC membrane ion-selective electrodes, the ratios of PVC, ionophore, plasticizer, and ionizer, as well as the types of plasticizers and ionizer, are critical parameters influencing the potentiometric performance of the electrode [6]. To identify the electrode with optimal potentiometric performance, various membrane compositions were prepared by altering these parameters. The potentiometric performance properties slope, detection limit, linear range, and R^2 value for the calibration curve of the electrodes fabricated using these membranes were systematically investigated.

Table S1. Membrane compositions were examined and studied for the purpose of optimizing membrane performance.

No	Composition of membrane, % (mg)						Slope, mV dec^{-1}	LRR	LOD X 10^{-5}	R^2
	Nonactin	PVC	PVC-COOH	NPOE	DOP	DOS				
1	3.0	30.0	-	67	-	-	12.6	1.0×10^{-4}-1.0×10^{-1}	1.0	0.939
2	3.0	30.0	-	-	-	67	45.9	1.0×10^{-5}-1.0×10^{-1}	1.0	0.999
3	3.0	30.0	-	-	67	-	19.1	1.0×10^{-5}-1.0×10^{-2}	1.0	0.864
4	0.8	32.8	-	-	-	67	26.4	1.0×10^{-5}-1.0×10^{-2}	1.0	0.955
5	1.4	31.6	-	-	-	67	34.5	1.0×10^{-5}-1.0×10^{-1}	1.0	0.954
6	2.0	31.0	-	-	-	67	24.3	1.0×10^{-5}-1.0×10^{-1}	1.0	0.947
7	3.5	29.5	-	-	-	67	15.6	1.0×10^{-4}-1.0×10^{-1}	1.0	0.932
8	0.8	-	32.8			67	44.1	1.0×10^{-5}-1.0×10^{-1}	1.0	0.998
9	2.0		31.0			67	35.6	1.0×10^{-5}-1.0×10^{-1}	1.	0.99
10	2.2		30.8			67	32.6	1.0×10^{-5}-1.0×10^{-1}	0.5	0.987
**11**	**3.0**	**-**	**30.0**	**-**	**-**	**67**	**58.2**	**1.0×10^{-5}-1.0×10^{-1}**	**0.7**	**0.998**
12	3.5	-	29.6	-	-	67	43.9	2.5×10^{-5}-1.0×10^{-1}	1	0.996

For the potential stability of the ammonium-selective RACNT and PGE, the slope was measured at regular intervals over 6 h. The change in slope per hour (slope/h) was recorded to evaluate the stability of both electrodes over time. Over the 6-hour period, the RACNT electrode exhibited a consistent decline in slope, with an average change of -1.4 mV/h, indicating relatively stable potential behaviour with a gradual decrease in sensitivity. In comparison, the PGE showed a more pronounced decline, with an average change of -3.8 mV/h, suggesting a faster loss of sensitivity over the same period.

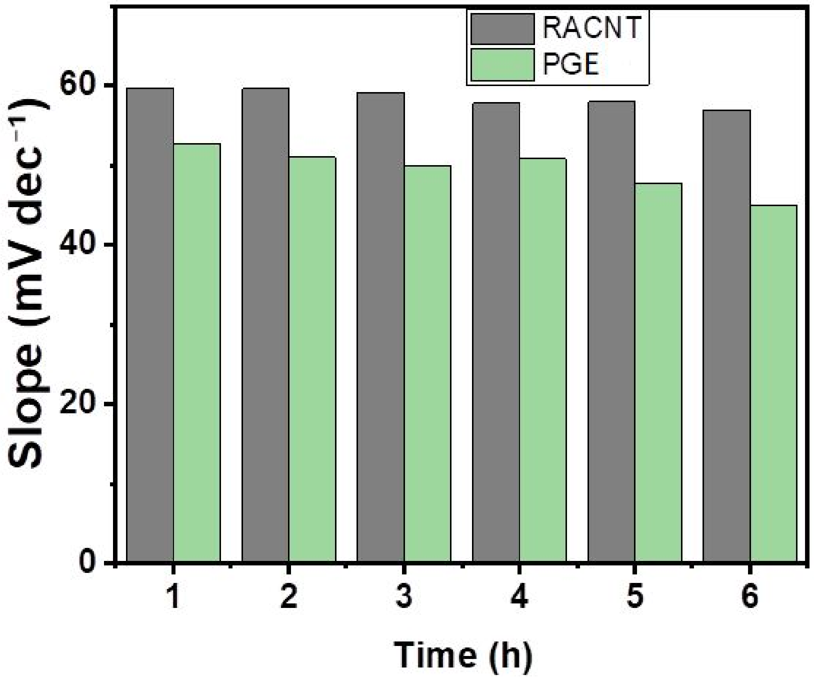

Figure S7: Change in the slope of the ammonium-selective electrodes with time (hour)

Determination of the lifetime of the ammonium-selective electrodes

To determine the lifetime of the ISE utilizing both RACNT and PGE, calibration curves were created by taking measurements on different days in NH_4^+ solutions within the concentration range of 1.0×10^{-5} to 1.0×10^{-1} M, where both ISE exhibited a linear response. Figure S8 reveals a decline in the slope of both the RACNT and PGE at ~fifteenth day. For commercial ISE, the lifespan is defined as the time taken for the ISE's slope to decrease to ~70% of its initial value. Accordingly, for the µISE, the initial slope (58.2 mV per decade concentration change) should decrease to 40.7 mV per decade concentration change by the end of the usage period. It was observed that after 15 days of use, the slope of the electrode reduced to ~38.7 mV per decade concentration change. As a result, the lifespan of both the RACNT and PGE could be estimated to be ~15 days. Before the measurements, the ISE were conditioned in a 1.0×10^{-2} M NH_4^+ solution for 30 min. When not in use, the ISE were stored in a closed and dark environment at room conditions.

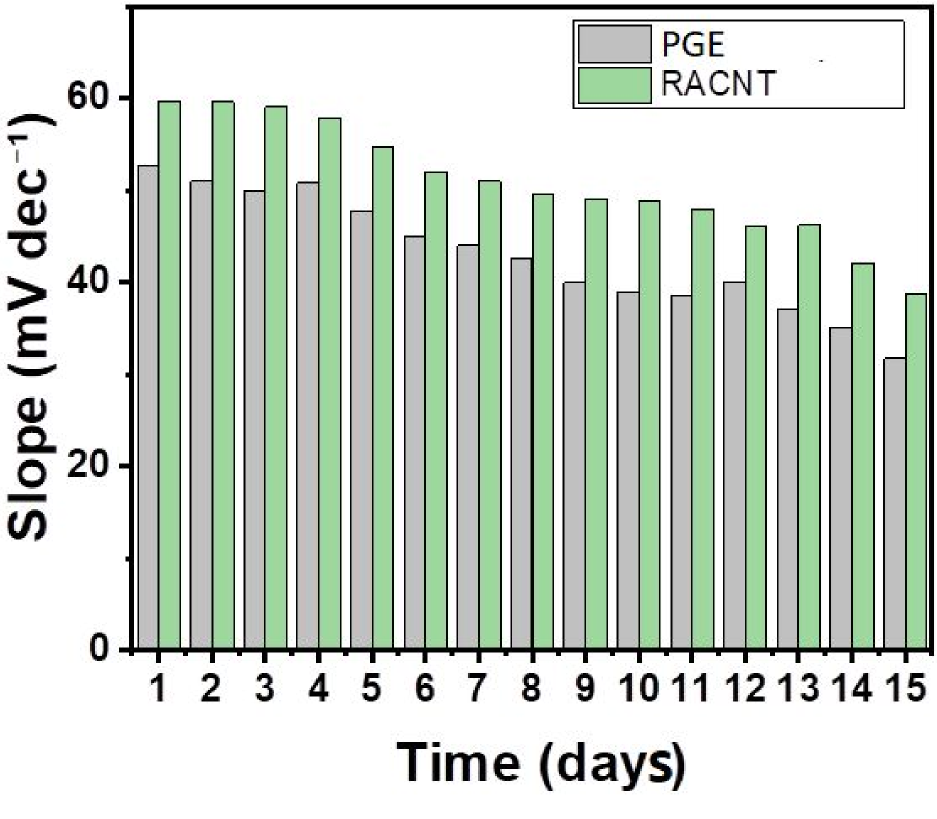

Figure S8: Change in the slope of the NH_4^+-selective ISE with time (days)

Comparison of solid contact type and K^+ selectivity NH_4^+–ISEs

A critical factor affecting the performance of NH_4^+-ISE is their selectivity towards NH_4^+ over K^+ ions, due to their similar ionic properties. In this context, we compared the NH_4^+-ISE developed using RACNT with the previous reports. The comparison focuses on solid contact types and their respective K^+ selectivity, expressed through selectivity coefficients. Table S2 summarizes the solid contact types used in NH_4^+-ISE and their K^+ selectivity.

Table S2. Comparison of solid contact type and K^+ selectivity for NH_4^+-ISE using nonactin ionophores

Type of solid contact material	$-K^{pot}_{NH_4^+,K^+}$	References
Inner-filling solution	0.6	[7]
Inner-filling solution	Strong K^+ interference	[8]
Solid-state (silver substrate)	0.8	[9]
Screen-printed electrode	1.4	[10]
Screen-printed electrode	1.2	[11]
Solid-state	1.8	[12]
Solid-state (GCE)	0.8	[13]
Solid-state (GCE)	0.9	[14]
Solid-state (GCE + PPy)	1.0	[15]
Pencil-draw graphite	0.65	[16]
Graphite paste	0.85	[17]
ISFETs with SiO_2/Ta_2O_5 gate insulators	1.2	[18]
Inner-filling solution	1.2	[19]
Solid-state (silver)	1.7	[20]
Solid-state	1.4	[21]
Solid-state (carbon tape)	0.8	[22]
**Solid-state (RACNT-GF)**	**1.8**	**Our work**

Contact angle measurements and XPS analysis for electrodes

Contact angle (CA) measurements were performed to characterise the surface properties of the RACNT as compared to the PGE. The CA measurements indicated that the RACNT exhibit a relatively more hydrophobic surface (CA is approximately 148°) compared to the PGE (119°), further supporting that the water layer formation is unlikely.

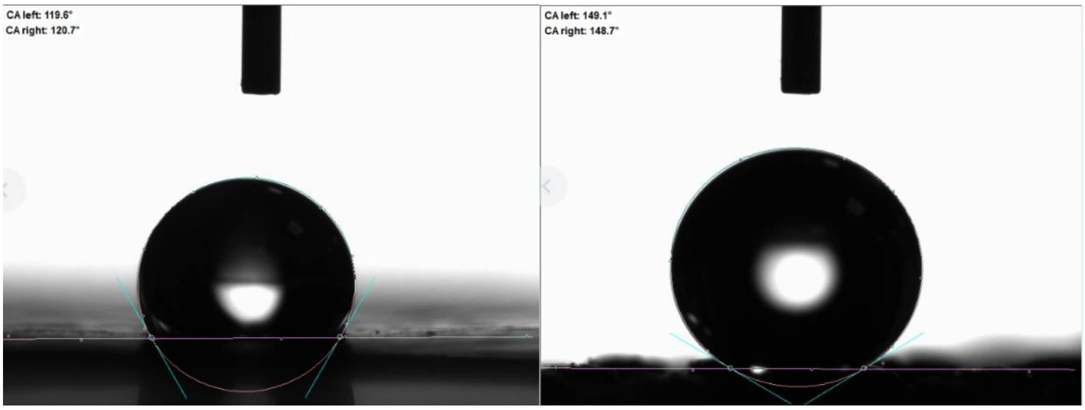

Figure S9 Water contact angle measurements of PGE (left) and RA-CNT (right)

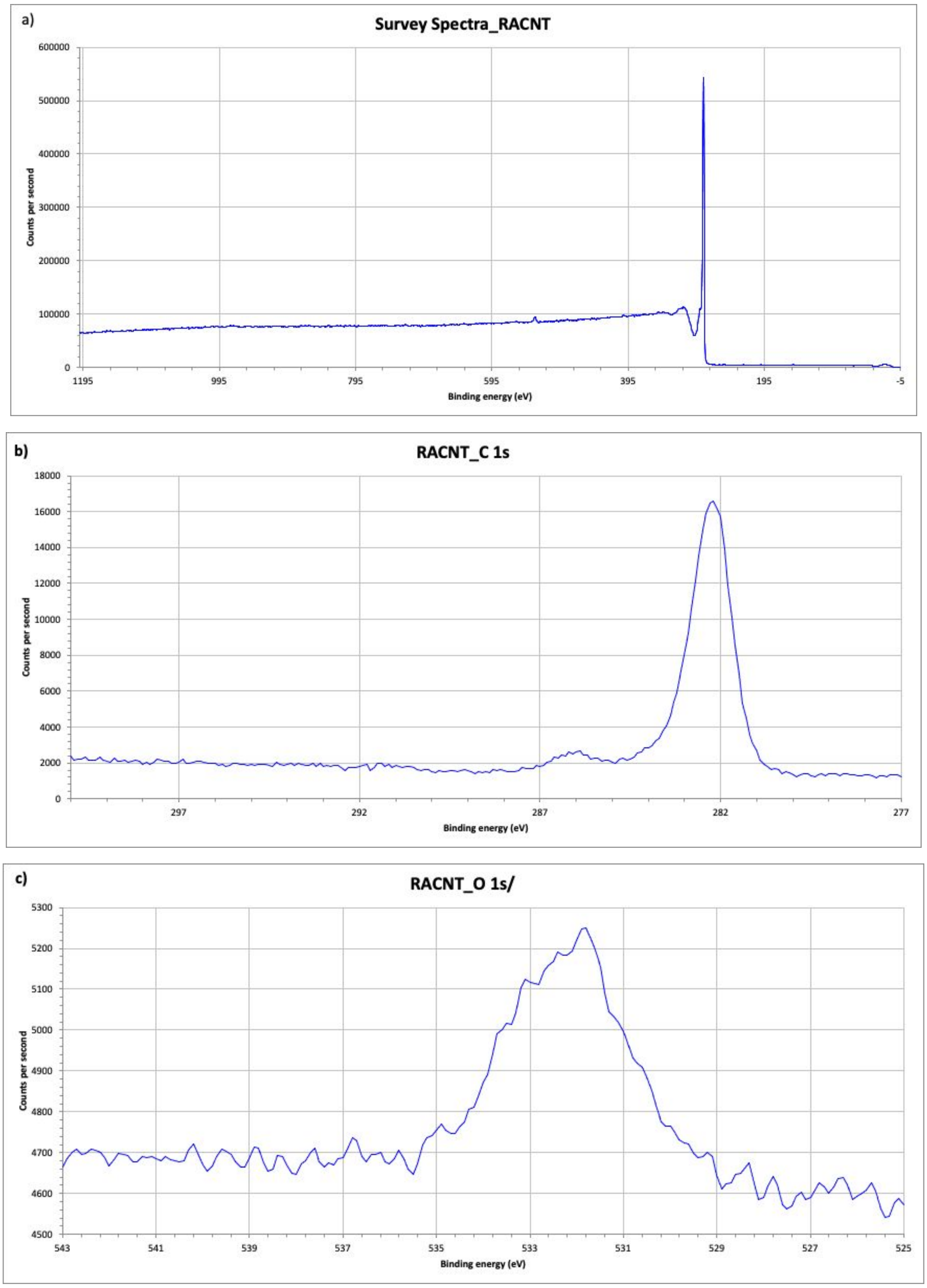

Figure S10: a) XPS spectra of RACNT, b) C1s spectra of RACNT, c) O1s spectra of RACNT

XPS analysis was performed at a pass energy of 50 eV. An Al Kα monochromatic (1486.68 eV) beam was used with a spot size of 200 µm (10 scans). As shown in Fig. S10, C1s spectra exhibited binding energy at 284.3 eV that corresponds to sp^2 hybridized carbon. The peak located at 285–286.5 eV is attributed to C=O bond from carboxyl groups. The O 1*s* region indicates binding energy at 532eV that corresponds to C=O bond from carboxyl groups. The reduced intensities of the oxygen peaks show that hydrophilicity of RACNT is marginal. XPS analysis concurs with CA measurements ascertaining that the water layer formation on RACNT is unlikely.

Potential Stability of Reference Electrodes

To verify the Potential Stability of the Reference Electrodes, potentiometric measurements were conducted using a series of standard Cl^- solutions. both the internal solution containing (Figure S11-A) and pseudo reference electrode (Figure S11-B). These solutions were prepared by diluting a 1.0×10^{-1} M potassium chloride stock solution with appropriate volumes of deionized water. The recorded potential values in response to varying chloride concentrations are presented in Figure S11. Reference electrodes containing saturated KCl exhibited a stable potentiometric response to increasing Cl^- ion concentration as expected. The pseudo-reference electrode without an internal solution demonstrated stable potential within the chloride concentration range of 1.0×10^{-6} to 1.0×10^{-3} M, but exhibited a response to chloride ions at concentrations as low as 1.0×10^{-2} M. This restricts the use of the pseudo-reference electrode without an internal solution in real samples with concentrations exceeding 1.0×10^{-2} M, particularly for chloride ions.

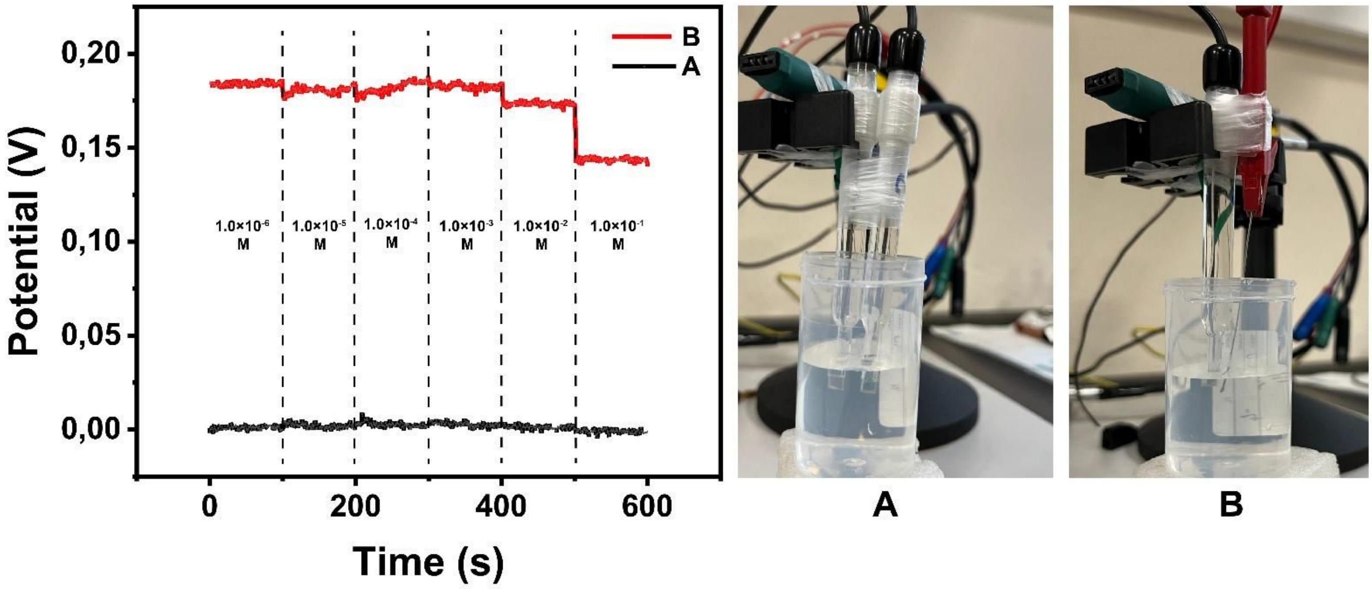

Figure S11: Potential stability measurements of reference electrodes

References

[1] A. Roussey, N. Venier, H. Fneich, L. Giardella, T. Pinaud, S. Tahir, M. Pelaez-Fernandez, R. Arenal, A. Mehdi, V. Jourdain, Mater. Sci. Eng. B Solid-State Mater. Adv. Technol. 2019, 245, 37.

[2] H. Murphy, P. Papakonstantinou, T. I. T. Okpalugo, J. Vac. Sci. Technol. B Microelectron. Nanom. Struct. 2006, 24, 715.

[3] C. Thomsen, S. Reich, J. Maultzsch, Philos. Trans. R. Soc. A Math. Phys. Eng. Sci. 2004, 362, 2337.

[4] A. D. Dobrzańska-Danikiewicz, D. Łukowiec, D. Cichocki, W. Wolany, Arch. Mater. Sci. Eng. 2013, 64, 103.

[5] S. Sakurai, H. Nishino, D. N. Futaba, S. Yasuda, T. Yamada, A. Maigne, Y. Matsuo, E. Nakamura, M. Yumura, K. Hata, J. Am. Chem. Soc. 2012, 134, 2148.

[6] Shamsipur, Mojtaba, et al. "A schiff base complex of Zn (II) as a neutral carrier for highly selective PVC membrane sensors for the sulfate ion." *Analytical chemistry* 73.13 (2001): 2869-2874.

[7] F. S. de Viteri and D. Diamond, Electroanalysis, 1994, 6, 9–16.

[8] A. Radomska, E. Bodenszac, S. Glab and R. Koncki, Talanta, 2004, 64, 603–608.

[9] C. Dumschat, M. Borchardt, C. Diekmann, J. Hepke, K. Cammann and M. Knoll, Fresenius'J.Anal. Chem.,1994, 348, 553–555.

[10] R. Koncki, S. Głąb, J. Dziwulska, I. Palchetti and M. Mascini, Anal. Chim. Acta, 1999, 385, 451–459.

[11] N. H. Chou, J. C. Chou, T. P. Sun and S. K. Hsiung, IEEE Sens. J., 2009, 9, 665–672.

[12] T. Guinovart, A. J. Bandodkar, J. R. Windmiller, F. J. Andrade and J. Wang, Analyst, 2013,138, 7031–7038.

[13] R. Athavale, I. Kokorite, C. Dinkel, E. Bakker, B. Wehrli, G. A. Crespo and A. Brand, Anal. Chem., 2015, 87, 11990–11997.

[14] L. Ding, J. W. Ding, B. J. Ding and W. Qin, Int. J. Electrochem. Sci., 2017, 12, 3296–3308.

[15] D. P. Quan, C. X. Quang, L. T. Duan and P. H. Viet, Environ. Monit. Assess., 2001, 70,153–165.

[16] J. Choosang, A. Numnuam, P. Thavarungkul, P. Kanatharana, T. Radu, S. Ullah and A. Radu, Sensors,2018, 18, 3555.

[17] J. Schwarz, K. Trommer and M. Mertig, Am. J. Anal. Chem., 2018, 9, 591–601.

[18] A. Bratov, N. Abramova, J. Muñoz, C. Domínguez, S. Alegret and J. Bartrolí, J. Electrochem. Soc., 1994, 141, L111–L112.

[19] A. Bratov, N. Abramova, J. Muñoz, C. Domínguez, S. Alegret and J. Batrolí, J. Electrochem. Soc., 1997, 144, 617–621.

[20] P. W. Alexander, T. Dimitrakopoulos and D. B. Hibbert, Electroanalysis, 1997, 9, 1331–1336.

[21] L. Y. Heng, S. Alva and M. Ahmad, Sens. Actuators, B, 2004, 98, 160–165.

[22] Y. Liu, R. Canovas, G. A. Crespo and M. Cuartero, Anal. Chem., 2020, 92, 3315–3323.
